# Humanities for medical students? A qualitative study of a medical humanities curriculum in a medical school program

**DOI:** 10.1186/1472-6920-6-16

**Published:** 2006-03-06

**Authors:** Caroline Wachtler, Susanne Lundin, Margareta Troein

**Affiliations:** 1 Lund University, Department of Clinical Sciences, General Practice/Family Medicine, Malmö University Hospital, SE 20502 Malmö, Sweden; 2 Lund University, Department of Ethnology, Finngatan 10, SE 223 62 Lund, Sweden

## Abstract

**Background:**

Today, there is a trend towards establishing the medical humanities as a component of medical education. However, medical humanities programs that exist within the context of a medical school can be problematic. The aim of this study was to explore problems that can arise with the establishment of a medical humanities curriculum in a medical school program.

**Methods:**

Our theoretical approach in this study is informed by derridean deconstruction and by post-structuralist analysis. We examined the ideology of the Humanities and Medicine program at Lund University, Sweden, the practical implementation of the program, and how ideology and practice corresponded. Examination of the ideology driving the humanities and medicine program was based on a critical reading of all available written material concerning the Humanities and Medicine project. The practice of the program was examined by means of a participatory observation study of one course, and by in-depth interviews with five students who participated in the course. Data was analysed using a hermeneutic editing approach.

**Results:**

The ideological language used to describe the program calls it an interdisciplinary learning environment but at the same time shows that the conditions of the program are established by the medical faculty's agenda. In practice, the "humanities" are constructed, defined and used within a medical frame of reference. Medical students have interesting discussions, acquire concepts and enjoy the program. But they come away lacking theoretical structure to understand what they have learned. There is no place for humanities students in the program.

**Conclusion:**

A challenge facing cross-disciplinary programs is creating an environment where the disciplines have equal standing and contribution.

## Background

Over the past 30 years there has been a trend towards the development of humanities curriculum in medical education, both in the United States and Europe. [1-3] Primarily, humanities researchers have developed the area of medical humanities, a discipline that is often part of a medical school faculty. Medical humanities can be defined as the application of the techniques of reporting, interpreting and theorising developed by the traditional humanities fields to phenomena within the traditional medical field [4].

The medical humanities can have both instrumental and non-instrumental functions in a medical school curriculum [5]. Humanities can have an instrumental function when directly applied to the daily work of the clinician. For example, study of visual arts has been used to improve the ability of the clinician to recognize visual clinical signs of disease in the patient. [6,7] Similarly, literature study has been used to train empathy and skills for handling ambiguity. [8-10] Likewise, the evaluation of case study narratives has been used to improve clinical skills [11]. The humanities have a non-instrumental function when they lead to general education, personal development, or new ways of thinking beyond the biomedical perspective. [4-6,8,10] For example, study of the humanities has been used to develop self-reflexivity and understanding of the role of the professional in society. [12,13]

Medical humanities programs that exist within the context of a medical school have certain existential problems, because the space created for these programs is defined by the medical school. Sometimes, an assumption is made that medical humanities curricula can, through their non-instrumental function, be used to make physicians more "humane" [2,8,13-15]. This assumption often occurs in the literature as an ideological motivation for the implementation of medical humanities programs. However, the equation of "humanities" with "humane" is awkward since the humanities disciplines consist of different schools of theory and research, not of one path to becoming a more caring person/physician. Alternately, the restriction of the humanities to their direct, instrumental, application in medical situations can limit the scope and possibilities for these disciplines within medicine [12,14,16]. Different medical schools make space for the medical humanities in different ways by defining curricular goals, and by implementing and examining the curriculum [17]. In the literature, problems that arise in establishment of a medical humanities program within a medical school are described. However, the way problems arise and why they arise has not been examined.

At Lund University in Sweden, the medical humanities became an explicit part of the medical curriculum in 2000 with the start of the Humanities and Medicine program (HumMed). This program involved teachers and administrators from the medical, humanities and theology faculties. The program was initiated and funded by the medical school faculty. It included elective courses offered to medical and humanities students, evening lectures open to the entire university, and independent study projects for students at both faculties.

### Aim

The aim of this study was to explore problems that can arise with the establishment of a medical humanities curriculum in a medical school program. To do this, we examined the ideology of the Humanities and Medicine program at Lund University, the practical implementation of the program, and how ideology and practice corresponded.

## Methods

### Theoretical perspective

Our theoretical approach in this study is informed by derridean deconstruction and by post-structuralist analysis. We assume that each stakeholder's representation of an event constitutes a construction of "truth" about what happened. Our focus is on how these different truths are constructed and on what the relationship between different truths about the same event can show us. [18-20] In this study, we examine the establishment of a medical humanities program by unpacking and comparing the different stories told in the official texts about the medical humanities program, in one of the author's field study of a medical humanities courses, and in interviews with students.

#### Ideology: The official story

Examination of the ideology driving the humanities and medicine program was based on a critical reading of all available written material concerning the Humanities and Medicine project. This included minutes from meetings, proposals, documents of faculty decisions, the website, course descriptions from the university catalogue, budget accounts, and local media. The project secretary, former and current project directors, and course directors were contacted with a request for this written material, and a search of the university homepage and of local newspapers was done.

#### Practice: One medical humanities course

The HumMed program offered four different elective courses to medical and humanities astudents. In autumn 2001 the course "Airbags for the Culture Crash" was given for the first time. (It soon became known as the "Airbags" course in student parlance.) For convenience' sake, this course was chosen as the subject of in depth study. Because of practical reasons, only this one course of the four could be studied, and an assumption was made that the other courses would be similar.

Before the course began, one of the authors (CW) contacted the two course directors and informed them of her interest in taking the course as a "participating observer." The course directors agreed, and CW presented herself as researcher and fellow student to the course participants at the first meeting. There she described the ethnographic methodology she would be using and that her goal was to document and aid understanding of what happened during the course. In addition, she explained that all participants in the course would be kept anonymous in the report.

The participatory observation method is commonly used in the social sciences, and is often associated with the fields of anthropology, ethnology and sociology. [21-23] The method entails doing fieldwork: taking part in the activities of a group and interacting with the members of that group, but at the same time observing and writing about those activities. The data produced from this activity are primarily field notes that describe events and conversations the researcher experienced. Field notes undergo formalized analysis after the researcher has finished her fieldwork. In this study, fieldwork included participation in all of the course meetings, literature reading and essay production that were part of the "Airbags" course.

#### Practice: Student voices

Two sets of in-depth individual interviews were done with five students from the "Airbags" course. Three of these students studied medicine, one woman and two men, and were about a third of the way through their studies. One of the students interviewed was a female undergraduate psychology student halfway through her studies. One of the students was a male undergraduate anthropology student.

Students were recruited for interviewing during CW's ethnographic study of the course. Recruiting was done three weeks into the course. CW announced to the class that she was interested in interviewing students in-depth about their experiences with the course. A total of 11 students expressed an interest. CW chose five of these on the basis of sex of the informants and what program they studied. Additionally, she chose students she hoped would represent both mainstream and outlier perspectives. This choice was based on the experience of hearing them discuss in class and from informal conversations. Later, interview texts were checked against material from informal conversations as recorded in fieldnotes to ascertain that this spread of perspectives was acquired.

The first set of interviews was done about halfway through the course and the second set was done six months after completion of the course. All interviews were held by CW in a room at the student union and lasted between 45 minutes and one and a half hour. Interviews were conducted using a broad topic guide. Discussion in the first set of interviews covered student motivation for taking the course, expectations, and current experiences of the course. Discussion in the second set covered what students thought of the course six months later, what experiences they remembered, and what had affected them. The interviews were taped and then transcribed verbatim, with grammar, pauses and interruptions preserved. Texts in quotes are translated direct citations from the interviews, and are numbered according to interview number: interviews A1-A5 are from the first set, and interviews B1-B5 are from the second set.

#### Analysis

We consider that the analytical conclusions we have drawn, or the "meaning" of our research, were generated and constructed in our interaction with the data [21,24]. The analytic methodology used in this study was based on de- and re-contextualisation of data by coding, categorizing, and memo writing [21,23]. This methodology is described by Crabtree and Miller as the "hermeneutic editing" approach to data analysis [25]. The data sets (official documents, field notes, interview transcripts), all of which were written texts, were analysed using this methodology. Chronologically, the data sets from the interviews and the field study were analysed first, and parts of this analysis were presented as written internal report. Two years later, the official document data set was collected and analysed. Finally, these different data sets were considered and analysed in relation to each other.

Coding was done in order to simplify and organize the data. CW read and re-read first for an overview of the body of material to identify general themes in the text. Then, texts were coded line by line by hand. These codes dissected out and named different, meaningful pieces of the text. Each code was one or two words that had a specific definition, and codes could be used multiple times, but were not used redundantly. The meaningful unit could be a passage, a sentence or a word which could be summarized, or named, by a code. Codes were not generated before the coding process, but the codes that were used were informed by the theoretical perspective described above. The actual code words used were often suggested by the text itself.

After coding the texts, CW inventoried the codes. Codes that were about similar ideas were grouped together in thematic categories. In this way, pieces of the texts that had themes in common could be examined in a new context. The categories were in turn grouped together, so a network of codes, subcategories and categories was created, each level implying more abstraction from the text.

During the coding and categorizing process, CW wrote theoretical memos as a way of describing and analysing the data. These memos are interpretative and the point of them was to describe the relationships between the codes, subcategories and categories as well as relationships between the categories. The memos were sorted and written up in the final stage of the analysis.

CW was the primary investigator and did the coding and categorizing independent of the other authors. CW and MT discussed the content of the theoretical memos in relation to the data. All three authors discussed the sorting and writing up of the memos. One author, SL, had personal experience with working on the project's board and as a course director. In discussing the analysis, we also discussed whether the conclusions fit with her experience. The analysis presented here is a result of the consensus of these discussions.

### Author backgrounds

CW was, at the time of the study, a medical student at the University of Lund and has a Bachelor's degree in cultural anthropology. SL is professor of ethnology and has been involved in the establishment of the Humanities and Medicine project through her work on the project's board and as a course director for one of the project's courses. She has also been involved in interdisciplinary research projects between the department of ethnology and the medical faculty. MT is a primary care physician, senior lecturer in general practice/family medicine, and course director for the professional development program at Lund University's medical school.

## Results

### Ideology: The official story

The HumMed program was conceived of as a "meeting place" for students and faculty members from the medical and humanities faculties. But from the beginning, there was an emphasis expressed on the "need" for humanities education in the medical curriculum. The first official draft of the idea was presented summer 2000, a proposal for initiation of HumMed at Lund University. The proposal had been written by a team made up of two professors from the humanities faculty and four from the medical faculty, at the behest of the university chancellor. The proposal was presented to the medical faculty. It stated:

"The areas that will be covered are of great interest for medical professionals and health care personnel. Need for education in these areas is great. There is a similar interest and competence in the humanities faculty." [2000-08-31]

Thereafter followed some areas the group thought should be covered and examples of different institutions from which instructors could be recruited. HumMed was started up and funded initially by the medical faculty. The program's board was composed of administrators and teachers from both the medical and the humanities faculties. The president of the board was a professor from the medical faculty. The program's secretary support came from the medical faculty.

From the beginning, the humanities faculty was less involved in administration and did not provide funding for the program. The humanities faculty provided the "competency" to fill a "need". The medical faculty provided funding, and students. Later, HumMed would be even more focused on filling a curricular need for the medical faculty. In 2004, HumMed's board did a survey of pedagogic elements already present in the medical curriculum that had a possible humanities aspect, and set an unofficial goal that every medical student take one humanities course.

The motivation for HumMed was presented in the local media. The program was considered valuable for medical students. The benefit, or involvement, for humanities students was not addressed.

"Physicians and technicians work today in a highly technological environment. However, in their professional roles they are often close to topics and problems that are tied to the humanities or theology. I felt there was a need to emphasize this through cooperation with the humanities and theology areas." (Professor Per Belfrage interviewed in Lunds Universitet Meddelar, a university magazine, 2001-01-25).

Simultaneously as this "need" for humanities education for medical students was stressed, the official documentation shows the interdisciplinary nature of this project. Two of five board members were from the humanities faculty. Members of both faculties designed HumMed activities, which were open to medical and humanities students. In 2003, an attempt was made to secure shared responsibility for the program's finances from the Humanities faculty. In addition, some of the official documents present HumMed as interdisciplinary, with humanities and medicine sharing the program's space equally. For example, the program goals as presented on the website:

" [The program] aims to create meeting places for the Medical, Humanities and Theology faculties' students and teachers . . . To deepen the humanistic perspective within the Medical Faculty, as well as the medical perspective within the areas of Humanities and Theology."

Here, the HumMed project is presented as a neutral interdisciplinary environment, separate from both faculties, where an equal relationship benefiting both parties can be maintained.

#### The ideology of the elective courses

HumMed invested greatest time and resources on the elective courses: "Text Study and Creative Writing," "Life, Love and Death – Worth Knowing in Health Care," "Airbags for the Culture Crash", and "Science and Reliable Experience – Knowledge from a Scientific Theory Perspective." Two or three elective courses have been given each of the five years HumMed has existed, and therefore almost all of the courses have been given multiple times.

The majority of students who attend the courses are medical students. The courses tend to be held at times that suit the medical student schedule, either as evening courses or during the medical student's elective period. Course information was not initially available in the university course catalogue, instead, it was available on the internet through the medical faculty website. Administratively, the courses are run by the medical faculty, although teaching is done by members of both the medical and humanities faculties.

The learning objectives for each course are shown in Table 1. In Life, Love and Death, "the biomedical and humanities 'double views' will both be expressed in relationship to human experience and problems." The Airbags course has a similar aim of "an historic as well as a current perspective on differences between the humanistic and natural science views on mankind and use of data". In both courses, familiarity with the "debate" between the medical and the humanities perspectives is a learning objective. In these texts, humanities and medicine are polarised, they are two opposite ("double, debating") views. This oppositional relationship between humanities and medicine is weighted. The humanities disciplines are expected to serve medicine. Both Life, Love and Death and Airbags have the use of humanities instruments in the medical setting as learning objectives. In Text Study and Life, Love and Death, the courses aim to "introduce a humanities perspective within medicine." All four courses assume that students will apply scientific tools traditionally used within the humanities fields to medical situations, but not the reverse. The humanities are to provide a service within the context of the medical perspective.

### Practice: One medical humanities course

The "Airbags" course was held for the first time during the fall semester 2001, open to both humanities and medical students. Thirty-two students took the course, of which 29 were medical students. In total there were 12 meetings over 12 weeks, each three to four hours in the evening, including a break with sandwiches provided. There were two course directors, a doctor who is a senior lecturer at one of the university hospitals, and a PhD student in ethnology (referred to as the Doctor and the Ethnologist from here on).

Completion of the course resulted in five Swedish credits, equal to one-fourth of a semester's studies, for both students from the humanities and the medical faculties. At Lund University, medical students must take a total of 10 elective credits. These credits may take to form of independent study or elective courses within or outside of the medical faculty. The "Airbags" course gave 5 elective credits for the medical students. Humanities students also received 5 elective credits for the course, which contributed in varying amounts depending on what degree program the student studied.

Generally, each evening started off with one or more lectures. Lecturers were from the medical faculty, the ethnographic and philosophic institutions, and from outside of the university. The Doctor gave three lectures and the Ethnologist gave two. Lectures had three concentrations. First, at the beginning of the course, were lectures situating students within the university. These defined the humanities, the Humanities and Medicine project, and the concept of education. Second, starting a few meetings into the course, were lectures about the construction of "We" and "the Other" and the concept of culture. Finally at the end of the course were lectures about culture clash and negotiation technique. After each lecture a discussion was held, either in small groups focused on specific questions or with the whole group directly after the lecture.

Attendance was mandatory for a passing grade. The course was assessed orally: students gave a presentation about an example of a culture clash they had experienced followed by a reflection, using concepts learned during the course.

Two parallel stories can be read in the field notes. First, there is a development in the thinking of the medical students that involves some utilization of concepts and skills from the humanities. Second, there is an unequal opposition between medicine and the humanities that is constructed and maintained during the course.

#### From right and wrong to nuances

Discussions moved during the course from being focused on what was "right" or "wrong" to a focus that allowed for the co-existence and examination of multiple perspectives. This movement can be seen in the discussions that started around an incident that occurred in the second meeting. The lecturer, a doctor, had in his talk repeatedly used the word "negro", and the phrase "any reasonable white man would know", when discussing PhD theses from the humanities faculty. One of the students had felt insulted but felt unable to question the lecturer; instead, she had gone to the Doctor after the meeting was over and reported her feelings. The doctor presented the problem for the class at the next meeting, and asked why no one had responded to the provocation, and a discussion started off, first tackling the question of whether or not the lecturer had done anything wrong:

The woman who had taken up the issue with the Doctor in the first place made herself known at this point in the discussion. "I thought it was an insult! I am black, and when he used that word I felt it was a racist attack."

A few other students raise their hands. They say they either didn't hear what the lecturer said, or that they heard it but that they didn't take it seriously. One man said at this point: "We are wasting our time by talking about this. It isn't a serious question, the lecturer is obviously not a racist, and what you are calling an insult has no basis in fact." (Fieldnotes; meeting 3)

The discussion continued, now focused on the appropriateness of the student's response. In this discussion, students tried to find answers to certain questions: Was it wrong for the lecturer to use that language? Was the student's response right? What were the "facts"? The aim of this discussion was to identify right and wrong. However, it was apparent that not all students had experienced the lecturer's comments in the same way. The meeting ended without one conclusion of "what really happened".

Two meetings later, the discussion of the incident continued. This time, students were divided into small groups to discuss the incident and try to answer the question: "what should you do when you feel provoked?" First, the discussions were a repetition of the opinions that had come up earlier. However, the discussion changed focus, and when students gathered as a large group again to go through each group's response, the focus of this large group meeting became nuanced. The questions discussed were: Why did this kind of problem occur, and what were the possibilities for solving the problems? Students discussed the power structures that allow for provocation to come up, and how belief systems play a part.

It wasn't possible to come to a conclusion about "what really happened", because group members had different answers to that question. Instead the incident was allowed to be ambiguous, and became instead the starting point for a more theoretical discussion. The group used concepts introduced under the lectures (the concept of culture, the construction of "the Other") to develop an understanding of the problem.

### Humanities versus medicine

An opposition between the humanities and medicine was constructed and maintained during the course. At the second meeting, a joint lecture was held by a respected professor from the medical faculty, and a professor of theology. First, the theology professor gave a short summary of the difference between humanism, a humanitarian, the humanities and humanity. Then, he gave a short talk about what the humanities and theology faculties can have for practical purpose.

The medical professor's lecture was focused on the differences between the humanities and medicine. At one point:

. . . [he] turns his attention to the other books on the table. It turns out one pile is a pile of PhD dissertations from the medical faculty, and the other from the humanities faculty. He shows us how the books from the medical faculty are all the same size, in sober colours ranging from beige to grey to black. They he shows us the books from the humanities faculty, "they look like more fun", and points out the different shapes and sizes, the many different colours and the pictures on their covers. He reads off some of the titles in a jokey way, adding comments like "who knows what this one's about". (Fieldnotes; meeting 2)

Later, in explaining the physical differences between the theses, he said:

"There is a predictable need for medical research, we know what it is good for. But research in the humanities reflects a need for knowledge, a need that happens before we know what the knowledge is good for." (Fieldnotes; meeting 2)

In this example, there was a silent assumption of a shared, medical, perspective. The humanities were defined for the students in a very basic way, but medicine was never defined. Medicine was the norm. When the speaker held up dissertations from the humanities departments and joked about their content with the (mostly medical) students in the room, the assumption was of a shared scientific perspective where drably designed medical dissertations had an understandable, serious purpose. This purpose was not examined, it is "predictable" and "we know what it is good for." The more "fun" humanities books were full of knowledge that wasn't "good for" anything – at least not yet. The theology professor's attempts to justify humanities research were not matched by any attempt to justify the stack of medical dissertations. The humanities were outside the norm, opposite and subordinate to it. That evening, and throughout the course, students got a good picture of how medicine looks at the humanities but not of how the humanities look at medicine.

### Practice: Student voices

The medical students were very positive about the course. They felt they had time for discussion and that they developed their discussion skills. Students gave examples of new concepts they had learned that they felt helped them to navigate discussions about cultural difference:

"It is fun to have a little background structure to think about things. That stuff about culture – that you can look at it in different ways, as a unit or as more variable and open to influence, that's an example. All of this was stuff I was interested in earlier. But the thing is, if you don't have concepts, if you don't have words, you can't discuss it in a sensible way." (A2)

Students had a complicated relationship to the concepts presented in the course. On the one hand, these concepts were important: "if you don't have concepts, you can't discuss it." At the same time, those concepts were undermined. They were "fun", and they were expressed as nothing new: "this was stuff I was interested in earlier."

"I thought it was very interesting to see the models presented by the Ethnologist, that there are models for that kind of thing . . . you can wonder why that is necessary, but it was fun to see that way of working" (A3).

A connection to the theoretical background to these "models" is missing. It is "fun" to learn about these models, they are different and interesting, but there is no context for understanding why they have been developed and therefore, they are empty of meaning.

After the course was over, medical students felt they had been changed by it. They used the discussion skills acquired in the course and felt they had grown personally.

"During the time I took the course, a lot happened with me. And I believe it was because of the course. It was easily the most worthwhile five points I have taken, because so much happened. Partly because I now have some thoughts, or concepts, to move with in different areas. I can't say anything specific right now, but it feels like I can discuss new things, after this". (B2)

The students know that they have learned something in the course, and they can even, in the interviews, show what it is that they have learned. They use examples of concepts from the course to illustrate their points, and they reflect on how they behave in discussion, how they react to difference. But at the same time they "can't say anything specific" about what the course was about. The structure providing the conceptual environment in which the discussions have grown is made invisible. The humanities are taken out of context and disarmed.

The interview data shows opposition and conflict between the humanities and medicine. The medical students saw the Airbags course as different from the rest of their medical education. While this was experienced as positive, the implication is that the humanities based course content was less serious that the rest of medical education (the word "fun" is used many times):

"We medical students, we want to work hard and show how serious we are, interested in science. But in the course everything was more vague. It was okay to take the discussion on another level. To maybe talk about things that hadn't been at all okay in a strict scientific environment". (B5)

The course was not "serious", "hard work", or scientific. The "vagueness" of this humanities segment in the medical student's environment may explain why students could not contextualise what they had learned.

Only three humanities students took the course, and one of them dropped the course halfway through. Comments from humanities students show that the role humanities served in the context of the course was not always positive:

"Their other studies, for those who study medicine, they are much more scientifically anchored than this course. So instead, they think 'oh, humanities, that's something you can meet over a beer and chat about.' . . I don't feel that the course in any way increased my understanding of cultural meetings. A better course title would have been 'Humanities for Medical Students."' (A1)

For this student, the medical frame to the course is obvious, and she is placed outside of it. The concepts from her scientific perspective are made into something to "chat about" and her expectations from the course are not addressed. She is made powerless and frustrated by the opposition constructed during the course.

### Ideology and practice

In this study, we see that the HumMed program was defined from a medical perspective. The ideological language used to describe the program calls it an interdisciplinary learning environment but at the same time a place where the medical student's "need" for "humanities" education can be fulfilled. The ideology of interdisciplinarity is undermined by a simultaneous demand on the usefulness of the summarily described "humanities" for the medical faculty. In practice, the "humanities" are constructed, defined and used within a medical frame of reference. Medical students have interesting discussions, acquire concepts and enjoy the program. But they come away lacking theoretical structure to understand what they have learned. Non-medical students hardly exist in the program. The humanities disciplines serve medicine. At the same time, the ideological definition of the program as "interdiscipliany" does not allow this medical frame of reference to be open for questioning.

Within the medical frame of reference, the humanities disciplines are positioned as the opposite of medicine. In this study, an examination of the practice of the HumMed program shows that the humanities disciplines are constructed as opposite and inferior: not-science, not-practical, not-academic, complementary to medicine, not equal to medicine.

A post-structural analysis shows that the construction of this opposition can serve a purpose. Through the dichotomy "Humanities and Medicine" constructed in the HumMed program, the vague "humanities" serve to define the borders of useful, practical, scientific "medicine". In this system, usefulness is valued, vagueness is not. The "humanities" give definition to medicine by being constructed as opposite, and in this opposition a power differential is also constructed. "Medicine" is dependent on the "humanities" for its status. If the humanities disciplines were no longer defined in the context of the medical perspective but instead defined themselves in an interdisciplinary environment, "medicine" would lose its definition and as a consequence, some of its power. An interdisciplinary environment could evolve, but in that environment, medicine would not be the only self-evident "science" anymore.

The medical faculty had the administrative and economical upper hand in the HumMed program. The medical faculty also defined the program's frame and purpose, and it follows that the practice of the program reflected the medical perspective. In this system, where the medical perspective was so self-evident, there was no room for the humanities disciplines to define what they wanted from medicine.

## Discussion

### This study and other research

Previous literature about the medical humanities has tended to focus on motivations for and risks with initiating such projects, philosophical issues, and reports of specific pedagogic efforts. This is the first study we are aware of that uses a systematic research strategy to understand how the practice of a medical humanities program worked.

Our results fit well with other discussions of medical humanities programs. A problem addressed in the literature is that the nature of the rationalist approach to knowledge taken by the medical perspective means that "students' thinking rarely moves (or is moved) outside simple dualisms; qualitative data or inquiry is implicitly (and often explicitly) positioned as the theoretical and methodological converse of quantitative data or inquiry" [26]. In addition, the goals of humanities and medical faculties differ within medical humanities programs. [27,28] As in our study, this can lead to opposition between the medical and humanities epistemologies.

It has been shown that some non-medical subjects, like cultural competency training, can be hidden in the medical school curriculum [29]. As in the present study, the ideology can differ from practice, and a similar power differential between non-medical and medical subjects can appear.

### Methodology

Ethnographic method is appropriate for generating explanations of phenomena that are directly relevant for the group being studied. [21-23] This kind of research can answer questions about why social processes work the way they do and can describe and explain experience. However, the conclusions drawn in this type of study are not final; instead they pose new questions and are always open to reinterpretation. In this study, the intention is not to make a generalisation about all medical humanities programs from our data set. We can draw conclusions only about what happened at this particular program and this particular course. However, this study raises questions about what can happen in the creation of medical humanities programs in medical school curricula. By showing how the unidirectional power differential is constructed and maintained at the program we have studied, we hope other educators will consider this issue in their own programs.

Interviews were only done with students from one course and only with a small number of individuals from the course. However, these interviews were judged to include a balance of mainstream and outlier perspectives when compared to informal conversations the investigator had with other course participants and recorded in field notes. Only one course was studied in depth. During the analysis, SLs experience was used as a check which allows us to say that the process seen in the Airbags course was probably similar to what happened even in the other three courses offered by the HumMed program. However, because of these limitations in this study, we have been cautious in extending our conclusions to include all of the elective courses given by the HumMed program. The conclusions describe a trend, not a rule.

The authors' backgrounds mean that two of them (CW and SL) approach the material having been members of the groups under study. Belonging to the groups at study can give useful knowledge about the relationships and structures at play in the groups, and it can ease contact with informants [22]. CW is herself a medical student and knew the other students in the Airbags course, which made recruitment to interviews easy.

However, there can be risk for bias when researchers belong to the groups at study. SL was involved in the creation of the HumMed program and sat for a period of time on its board. SLs personal experience of representing the humanities faculty in the HumMed context agreed with the analysis done in this study. As discussed above, SL's personal experience could be used as a check on the analysis. However, we consider that this experience did not bias the analysis, since the other two authors were also active in the analytical discussion

Data collection and analysis have been described in this study as two separate processes. However, we consider that since the researcher is the analytical tool, analysis is continuous during data collection. This informs what the researcher focuses on and the questions she asks [21]. In this study, this means our analysis of the data can be considered to be relevant for our research aim and for the group we studied. More general conclusions about the results are tentative.

Methods of assessing validity common to quantitative work are not applicable in inquiry-based investigations [30]. Trustworthiness of results can be improved, as in this study, by use of known methodology and by transparency in methodological and analytical description [23,30]. Using multiple researchers in evaluation and analysis of transcribed material can also add to the trustworthiness of results [31].

## Conclusion

A challenge facing cross-disciplinary programs is creating an environment where the disciplines have equal standing and contribution. We saw the intention for equality in the program's ideology, but already from the outset this was contradicted by the program's focus on medical student need, and the power relationship between the medical and humanities faculties that this reflected. This power differential made impossible the construction of a truly interdisciplinary environment, where equal representation and interaction of different disciplines gives rise to a "larger methodological picture" with new ways of doing research and solving problems [1].

This study indicates practical possibilities. Lifting the program to university level could be a way to avoid some of the problems seen. Creation of an autonomous medical humanities department, with a professorship and the status granted by a research budget, could be one way to do this. Another approach could be sharing the economic and administrative responsibility for the program equally between the faculties so that the power inherent in this responsibility is also shared. We have seen that the courses have been mainly created for medical students, and medical students have been the major users of this program. It could help to widen the scope of these courses, so that they are equally accessible to humanities and medical students, by changing course plan focus and adapting course times to fit both schedules.

This study makes explicit the possibilities of a medical humanities program. Opportunities arise from the access to shared curricular space. Within each course plan is a discussion of and awareness that two different approaches do not have to be two opposite approaches. In practice, students enjoy the program, there is utilization of new concepts, and students feel they grow. Introducing an element of reflexivity, where students and course directors, examine and make visible their own theoretical grounding and assumptions, could minimize the oppositioning we have seen.

Finally, introduction of the medical humanities field into medical education is a current trend. A critical awareness, examination and discussion of the power differentials that can arise during this process would help create more equal "meeting places" for the humanities and medicine.

## Competing interests

The author(s) declare that they have no competing interests.

## Authors' contributions

All authors participated in planning the study. CW did all data collection, analysed the data, and drafted the manuscript. SL and MT contributed to data analysis as described in the text, and were involved in revision of the manuscript. All authors read and approved the final draft of the manuscript.

## Pre-publication history

The pre-publication history for this paper can be accessed here:



## Figures and Tables

**Table 1:** Learning objectives from Humanities and Medicine courses.

**Text Study and Creative Writing**
• Illumination of historical problems through analysis of central works of fiction.
• Knowledge about a selection of central literary works' cultural and literary background.
• Orientation to the perspective and methods of literary theory.
• Development of creative writing skills with literature reading as background.
• A complementary goal for all education within the humanities is the ability to correctly use the Swedish language in speech and writing.

**Life, Love and Death**
• Development of knowledge of human experience so that more meaningful consultations with patients can be held and the quality of health care improved.
• Development of a "double view" on human problems.
• Familiarity with the main arguments in the current debate in the sphere where medicine and the humanities meet.

**Airbags for the Culture Clash**
• A safer platform to stand on in meeting with other cultures.
• Development of the ability to feel curiosity and openness when meeting difference.
• Development of the ability to put oneself in another's place and speak another's opinion.
• Ability to lead a simple negotiation.
• Familiarity with the debate on education that is held around the subject of college education.
• Familiarity with the ideas behind the Humanities and Medicine project.

**Science and Reliable Experience**
• Development of an inclination to ask the question "How does one know?"
• Development of a critical position to individual knowledge.
• Acquaintance with basic concepts, ways of thinking and methods within the different sciences and under different epochs in order to create understanding of scientific diversity and to stimulate cross disciplinary research.
